# Changes in Reported Secondhand Smoke Incursions and Smoking Behavior after Implementation of a Federal Smoke-Free Rule in New York State Federally Subsidized Public Housing

**DOI:** 10.3390/ijerph19063513

**Published:** 2022-03-16

**Authors:** Laurel E. Curry, Ashley L. Feld, Todd Rogers, Ellen M. Coats, James Nonnemaker, Elizabeth Anker, Christina Ortega-Peluso, Haven Battles

**Affiliations:** 1 Center for Health Analytics, Media, and Policy, RTI International, 701 13th St. NW, Washington, DC 20005, USA; 2 Center for Health Analytics, Media, and Policy, RTI International, 3040 East Cornwallis Road, Research Triangle Park, Durham, NC 27709, USA; afeld@rti.org (A.L.F.); trogers@rti.org (T.R.); ecoats@rti.org (E.M.C.); jnonnemaker@rti.org (J.N.); 3 New York State Department of Health, ESP Corning Tower, Room 1055, Albany, NY 12237, USA; elizabeth.anker@health.ny.gov; 4 New York State Department of Health, ESP Corning Tower, Room 1072, Albany, NY 12237, USA; christina.peluso@health.ny.gov (C.O.-P.); haven.battles@health.ny.gov (H.B.)

**Keywords:** public policy, secondhand smoke, anti-smoking

## Abstract

This study assessed changes in smoking behavior and secondhand smoke (SHS) exposure after implementation of the U.S. Department of Housing and Urban Development (HUD) rule prohibiting the use of cigarettes, cigars, pipes, and waterpipes in all federally subsidized public housing, including within residential units (apartments). Using quantitative data from a repeated cross-sectional mail survey of New York State residents of five public housing authorities (N = 761 at Wave 1, N = 649 at Wave 2), we found evidence of policy compliance (99% decrease in odds of self-reported smoking in units, OR = 0.01, *p* < 0.01, CI: 0.00–0.16), reduced SHS incursions (77% decrease in odds of smelling smoke within developments, OR = 0.23, *p* < 0.01, CI: 0.13–0.44), and lower reported smoking rates in July 2018 (9.5%, down from 16.8%), 10 months after implementation of the rule. Despite evident success, one-fifth of residents reported smelling smoke inside their apartment at least a few times per week. This study provides insights into how the policy was implemented in selected New York public housing authorities, offers evidence of policy-intended effects, and highlights challenges to consistent and impactful policy implementation.

## 1. Introduction

Exposure to secondhand smoke (SHS), smoke from burning tobacco products or that has been exhaled, exacerbates asthma and other respiratory conditions, causing lung cancer, stroke, and heart disease [1]. Smoke infiltration among residential units in close proximity, which is prevalent in multi-unit housing apartment complexes, puts residents of those units at particular risk for the harms of SHS exposure [2]. Levels of SHS exposure are highest among people living in poverty and those living in rental housing [3]. People who live in public housing face increased risk of exposure to SHS, because smoking prevalence is relatively higher among those with lower income [4]. Approximately 10.4 million people live in subsidized housing in the United States [5]. Many of those residents may be particularly vulnerable to the effects of SHS because a large proportion of them are children (36%), elderly (35%), or people with a disability (39%), and may have limited ability or resources to find housing where they are protected from exposure to SHS [6]. 

On 5 December 2016, the U.S. Department of Housing and Urban Development (HUD) published a rule that prohibits use of cigarettes, cigars, pipes, and waterpipes in all federally subsidized public housing to benefit the health of public housing residents, visitors, and staff; improve indoor air quality; reduce the risk of fires; and decrease maintenance costs [7]. The rule requires entirely smoke-free apartment buildings and offices and a minimum outdoor 25 foot buffer zone. Importantly, the rule prohibits smoking within all 900,000+ public residential units (apartments) in the United States [6]. The rule took effect on 3 February 2017, but allowed an 18 month window for Public Housing Authority (PHA) implementation (i.e., by no later than 30 July 2018). PHAs had the options to restrict smoking further to dedicated outdoor smoking areas, make their entire grounds smoke-free, and/or include a prohibition on use of electronic nicotine delivery systems (ENDS) such as e-cigarettes. 

Limited information exists about the effectiveness of smoke-free multi-unit housing policies [8], and even less is known about the implementation and impact of the HUD rule specifically. Several case studies report on voluntary smoke-free policies in multi-unit housing implemented before the HUD rule [9,10,11,12,13,14]; however, we are aware of only two published evaluations of the HUD rule. In the first, Plunk [15] found an immediate reduction in both airborne nicotine and particulate matter in the month following implementation of the smoke-free rule in six federally subsidized public housing buildings in Norfolk, Virginia. A year later, however, both markers of ambient SHS had increased [15]. These findings imply initial compliance with the rule followed by longer-term noncompliance and a possible increase in indoor smoking. The second available evaluation of the HUD rule found no effect on air nicotine levels or particulate matter in nonsmoking apartments in addition to no change in cigarette butt counts in stairwells and hallways in a sample of ten New York City Housing Authority (NYCHA) buildings compared to a sample of Section 8 public housing buildings not subject to the rule [16]. 

In New York State, more than 10 million people (51% of the state population) live in multi-unit housing [2], including those living in nearly 200,000 public housing units [6]. Prior to HUD rule implementation, many New York PHAs had already implemented extensive smoke-free policies, but others, including the 173,762-unit NYCHA, the largest PHA in the nation, had yet to adopt comprehensive smoke-free policies [17]. This study sought to understand the impact of the HUD rule on self-reported smoking behavior and SHS exposure among New York residents living in public housing.

## 2. Methods

### 2.1. Sample

We conducted repeated cross-sectional telephone interviews of PHA executive directors (EDs) and mail surveys of development managers (DMs) and residents in New York State using a clustered sample design. Although this report focuses on the resident survey, insights from the ED interviews will help contextualize these findings. For each of five regions of New York, we created a randomly ordered list of all PHAs that in 2017 did not yet prohibit smoking within apartment units. (The New York Tobacco Control Program defines five regions for their work: Metro (excluding NYC), New York City, Capital, Central, and Western.) With the exception of New York City (for which NYCHA is the sole PHA), we attempted to recruit the first PHA on the list in each of the other four regions using lead letters and telephone calls to EDs, moving down the sampling list if EDs did not respond or declined to participate. We sampled the same five PHAs at Wave 1 and Wave 2: NYCHA (New York City), Newburgh (non-NYC Metro), Schenectady (Capital), Cortland (Central), and Jamestown (Western). Because more than 80% of New York State’s public housing units are in NYC, we selected 16 developments that are administered by NYCHA and one development in each of the non-NYC regions for a total of 20 public housing developments across the state. Since the non-NYC PHAs were generally smaller, we had to randomly select multiple developments for three of them to achieve a target of 125 units within each PHA. In each of the 20 developments, we randomly selected 125 residential unit addresses, or selected with certainty all residential unit addresses in a development with fewer than 125 units. Per our participation agreements with the PHAs, we are not reporting PHA-level data from this analysis.

### 2.2. Procedure

From July to September 2017, 13–15 months before the final implementation date for the HUD rule, we conducted a Wave 1 survey of residents of five New York PHAs, including NYCHA, where residents were still allowed to smoke in their units. We conducted a Wave 2 survey from March to June 2019, approximately 9–11 months after implementation was required. In advance of sending the DM and resident survey packets, we conducted telephone interviews with the same five PHA EDs at both points in time. ED interviews covered current and upcoming smoke-free policies, education and enforcement activities, and ED beliefs and attitudes related to smoke-free public housing. DM and resident survey packets were mailed in English and Spanish, and residents received a USD 2 pre-incentive with the first mailing, which was followed by a reminder postcard and, if unresponsive, a second packet. The Wave 2 resident surveys were mailed to the “current resident” of the exact same addresses included in the Wave 1 sampling frame (not necessarily the same people), with a few exceptions:

- In Schenectady, we replaced 33 addresses because of two demolished buildings.
- In Jamestown, we did not send surveys to the 32 units at one complex, because implementation of the smoke-free policy was not required in that development.
- In NYC, we replaced one development that was converting to Section 8 housing and thus not subject to the HUD rule.

At Wave 1, we received nine DM surveys (50%) and 761 resident surveys (30.4% of all units in the sample). At Wave 2, we received 10 DM surveys (52.6%) and 649 resident surveys (26.3%). Survey materials requested that the same person take the Wave 2 survey, if possible. Of the Wave 2 respondents, 351 residents (57.4%) reported being or living with the same person who filled out and returned the Wave 1 survey in 2017. Five DMs (50.0%) reported being the same person who filled out and returned the Wave 1 survey in 2017. The RTI and NYSDOH Institutional Review Boards determined that this activity was conducted for surveillance and evaluation and thus did not meet the definition of research with human subjects. A letter accompanying the anonymous resident survey explained that participation was voluntary, questions could be skipped, and that information would be kept confidential to protect respondents’ privacy. Residents could keep the USD 2 cash incentive included in the survey packet, regardless of whether they returned the survey.

### 2.3. Measures

The resident survey asked questions on age (“What is your age?” (18–24, 25–24, 35–44, 45–64, and 65 or older)), sex (“Are you male or female?”), ethnicity (Latino or Hispanic origin), and race (White, Black or African American, Asian, Native Hawaiian or Pacific Islander, and/or American Indian or Alaska Native). Respondents were coded as multi-race if they selected more than one race response. The resident survey also asked about combustible tobacco use status (“Do you now smoke tobacco (cigarettes, cigars, pipes, or hookah) every day, some days, or not at all?”), vape use (“Do you now use electronic vapor products (e-cigarettes, e-hookah, vape pens) every day, some days, or not at all?”), and length of time in current apartment unit (“How long have you lived in this apartment unit?” (less than 1 year, 1 to 5 years, 6 to 10 years, more than 10 years)). All analyses were conducted on the subset of respondents who reported living in their unit for one year or more, in order to ensure that our analysis included only those Wave 2 participants who had lived in the development both before and after smoke-free policy implementation. Current tobacco use and current vaping were defined as smoking or vaping “every day” or “some days.” 

Repeat respondents. At Wave 2, 351 (57.4%) residents reported that they were the same person who filled out and returned the Wave 1 survey in 2017 (*n* = 336) or that someone else in their household filled out the Wave 1 survey (*n* = 15). Of those, 69% (*n* = 242) were confirmed to be repeat respondents on the basis of the presence of a complete survey from the respondent address at Wave 1. Models include an indicator for these “repeat respondents,” who were confirmed members of a participating household at both Wave 1 and Wave 2, to account for differences in responses between those who lived in their unit at both time points and those who may not have lived in the unit at both time points.

Quitting/cutting-down. At Wave 2, we asked residents, “Have you cut down, quit, or changed how you use tobacco products since July 31, 2018?” Response options were “Yes, I cut down on how much I use,” “Yes, I quit smoking,” “Yes, I changed what product I use,” “No,” and “I was not a smoker before the change in policy, and I’m not a smoker now.”

We measured self-reported SHS exposure in two ways: smelling smoke, and smoke incursion. 

Smelling smoke. We asked respondents “In the past 7 days, have you smelled tobacco smoke in each of the following areas of your apartment complex?” with options “Yes,” “No,” and “Don’t have this area” for each of the following: “Indoor shared areas, like stairwells and hallways,” “Shared laundry rooms,” “Lobby and/or lounge area,” “Recreation room and/or party room,” “Within 25 feet of your building,” “Shared large outdoor areas like parking lots, lawns, or playgrounds,” “Outdoor porches or patios,” and “Inside your apartment.” We coded respondents as having SHS exposure if they reported smelling smoke in at least one location. We also generated an additive index reflecting the number of places respondents reported smelling smoke in their development. This index ranged from 0 to 8, where 0 indicates a respondent did not smell smoke in any area and 8 indicates a respondent smelled smoke in each of the areas listed in the question. In defining this index, a respondent was treated as not having smelled smoke in an area if their development did not have that area.

Smoke incursion. We coded respondents as having smoke enter their unit if they responded to “In the past 6 months (Wave 1)/Since July 31, 2018 (Wave 2), how often has tobacco smoke entered your apartment unit from somewhere else in or around your building?” with “Every day,” “A few times a week,” “A few times a month,” or “Once or twice.” 

Noncompliance with the smoke-free policy was also measured in two ways: in-unit smoking by respondents, and in-unit smoking by someone else in the respondents’ apartment. 

In-unit smoking by respondent. We identified respondents who smoked inside their unit as those reporting one or more days in response to “During the past 7 days, on how many days did you smoke tobacco in your apartment unit?” This outcome was analyzed among current smokers, defined as those responding either “Every day” or “Some days” to “Do you now smoke tobacco (cigarettes, cigars, pipes, or hookah)?”

In-unit smoking by someone else in respondent’s apartment. We identified respondents experiencing in-unit smoking by someone else as those responding one or more days to “During the past 7 days, on how many days did someone else smoke tobacco in your apartment unit?”

Finally, we asked respondents about their own protective home smoking rules, another indicator of policy compliance. We coded respondents as having protective home smoking rules if they responded “Never” to “Inside your unit, how often is smoking allowed?”

### 2.4. Analyses

We present descriptive results for outcomes of interest at Wave 1 and Wave 2 among respondents who reported living in their current unit for 1 year or more (N = 726 (96.1%) at Wave 1, 619 (96.4%) at Wave 2). 

Among the same group of respondents, we used logistic regression to model binary outcomes of interest (smoke incursions, in-unit smoking, in-unit smoking by someone else). We used linear regression to model the number of places respondents reported smelling smoke in their development. All models accounted for within-unit correlation and controlled for PHA-level fixed effects. An indicator for Wave 2 (responses from the Wave 2 survey) served as the main independent variable to examine whether changes over time were statistically significant. Models also controlled for age (age 65+ vs. else), sex (male vs. female), current smoking (excluded from the model of in-unit smoking by respondent, which was restricted to only smokers), and repeat respondents (those who were residents of a participating household at both Wave 1 and Wave 2). 

All analyses were conducted using Stata 16 statistical software (StataCorp LLC, College Station, TX, USA).

## 3. Results

### 3.1. Sample Characteristics

We found no notable differences between Wave 1 and Wave 2 in sample composition (Table 1). All of the following analyses are restricted to the sample of respondents at each time point who reported living in their unit for at least one year at Wave 1 and Wave 2, which was the majority of the sample at each time point (N = 726 (96.1%) at Wave 1, 619 (96.4%) at Wave 2). 

### 3.2. Changes in Tobacco Use

A total of 23.7% of the respondents who used tobacco prior to the policy implementation reported cutting down on how much they used tobacco products since July 31, 2018, while 30.6% reported having quit smoking completely. The proportion of respondents reporting current cigarette, cigar, pipe, or hookah smoking (Table 2) was 16.8% at Wave 1 and 9.5% at Wave 2. 

### 3.3. Tobacco Use in Residential Units (Compliance)

The mean number of days smokers reported smoking in their unit within the past 7 days was 4.0 at Wave 1 and 2.3 at Wave 2 (Table 2). The mean number of days individuals other than the respondent smoked within a respondent’s unit was 0.4 at Wave 1 and 0.2 at Wave 2. The frequency of within-unit use of vaping products by residents who use these products was similar at the two time points, as was the frequency of others using vaping products within resident units. 

### 3.4. Smoke Incursions/SHS Exposure

**Figure 1:**
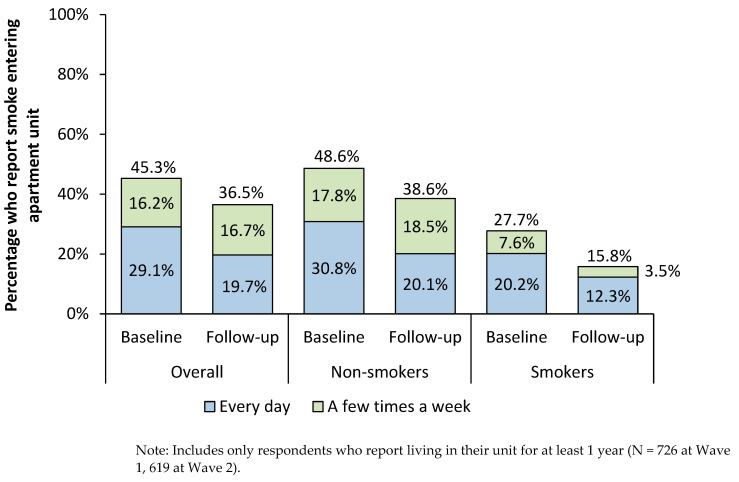
Proportion of respondents reporting smoke incursions every day or a few times a week, Wave 1 vs. Wave 2.

At Wave 2, a smaller percentage reported smoke entering their unit every day compared to the base period (Figure 1); however, 36.5% of residents still reported smoke incursions every day or a few times a week. Non-smoking residents reported greater frequency of smoke incursions than smokers. At Wave 2, 38.6% of non-smokers reported smoke entering their unit every day or a few times a week compared with 15.8% of smokers. 

The percentage of respondents who reported smelling smoke in any area of their building was 86.1% at Wave 1 and 73.0% at Wave 2 (Table 3). Decreases were also observed in the percentage of respondents who smelled smoke in each individual area. 

Logistic regression results in Table 4 indicate that respondents were at decreased risk of SHS exposure at Wave 2. Specifically, for Wave 2 respondents, the odds of smelling smoke anywhere within their development were 77% lower than those of Wave 1 respondents (OR = 0.23, *p* < 0.01). The odds of reporting smoke entering their unit for Wave 2 respondents were 61% lower than those of Wave 1 respondents (OR = 0.38, *p* < 0.01). Furthermore, linear regression results indicate Wave 2 respondents reported smelling smoke in 0.70 fewer places than Wave 1 respondents (*p* < 0.01). Those aged 65 or older reported smelling smoke less frequently and in fewer places than younger respondents (*p* < 0.05), and current smokers reported smelling smoke in fewer places than non-smokers (*p* < 0.05).

Wave 2 respondents were more likely to comply with the smoke-free policy. The odds of current smokers at Wave 2 reporting smoking in their unit were 99% lower than those of Wave 1 smokers (OR = 0.01, *p* < 0.01). The odds of respondents reporting someone else smoking in their unit were 55% lower at Wave 2 than at Wave 1 (OR = 0.45, *p* < 0.05) (Table 4). 

The odds of having restrictive home smoking rules at Wave 2 were 3.63 times the odds of having such a rule at Wave 1 (*p* < 0.01) (Table 4). Respondents who were aged 65 or older or male more often reported having restrictive home smoking rules than those who were younger or female, respectively (*p* < 0.05). Meanwhile, similar rules were less common among current smokers compared with non-smokers (*p* < 0.01). 

## 4. Discussion

Using data from a repeated cross-sectional mail survey of New York State residents of five public housing authorities, we found evidence of resident compliance with the HUD smoke-free rule and reduced SHS exposure. Ten months after implementation, we found (1) more residents reported never allowing smoking inside their unit, (2) fewer residents reported smoke entering their unit every day or smelling smoke in the past 7 days in living and common areas, (3) fewer residents reported using combusted tobacco “every day” or “some days,” and (4) nearly one-third of ever-smokers reported quitting smoking because of the policy change.

Although the average number of days smokers used tobacco in their unit decreased by half from Wave 1 to Wave 2, in-unit use of *vaping* products did not change, either among residents or their guests, nor did use of vaping products by residents generally. The HUD rule provided PHAs with discretion over whether to include ENDS in their smoke-free policies [7], and by the time of the Wave 2 survey, two of five PHAs had voluntarily included ENDS products in their smoke-free policy. In contrast, the decrease in number of smoking days among smokers and the reported prevalence of quitting because of the policy change suggests the rule had an impact on smoking behavior. Although not a direct comparison to our measure of cessation associated with implementation of smoke-free policy, self-reported quit rates also increased dramatically after a voluntary policy that included in-unit restrictions in a Portland, Oregon study of subsidized housing residents (average annual rate pre-policy: 2.6% vs. post-policy: 14.7%) [12]. The study also found that about half of the smokers reported cutting down, and the majority of those that cut down or quit said it was in part or wholly because of the policy [12]. 

The improvement in reported SHS incursions in our study is at odds with two published evaluations of the HUD smoke-free rule, which found little evidence of successful improvement in air quality before versus 12 months after policy implementation in Virginia and NYCHA [15,16]. It is difficult to compare our results with those of other studies due to the differences in study methodologies and measurement. Our evaluation approaches varied significantly: the Virginia and NYCHA studies used active and passive monitoring to measure air quality changes (nicotine concentration and PM_2.5_ concentration), whereas our study relied on self-reported incursions. The variation in outcomes may also be attributable to differences in the sampling mix: our sample included residents of non-NYCHA PHAs. Differences in policy implementation between the PHAs across state regions and between buildings within a region (i.e., within NYCHA) could also be at play. 

Several factors that arose in interviews with EDs of the five PHAs under study may explain the evidence of success of HUD rule implementation in New York State demonstrated in this study. First, all five PHAs provided tenants with resources to quit smoking as part of their implementation of the rule in the form of referrals to the Quitline and on-site cessation groups. Most PHAs also reported conducting awareness-raising activities—such as resident meetings and partnering with resident councils—to foster support for policy change among residents. In addition, the New York State Department of Health-funded grantees also offered both policy and cessation support to public housing staff and residents throughout the state. The Advancing Tobacco-Free Communities program worked with public housing authorities in the state to assist with drafting policies, plan and conduct resident meetings, provide educational materials and opportunities, and seek out opportunities to highlight the HUD policy change using both paid and earned media. The Health Systems for a Tobacco Free New York program also worked to educate local healthcare providers to prepare them to assist HUD residents with complying/quitting as necessary before, during, and after implementation.

Despite the evident success of HUD rule implementation in our sample of New York State PHAs, a year after the rule went into effect, one-fifth of residents still reported “smelling smoke” inside their apartment in the past seven days and more than one-third reported smoke entering their unit “from somewhere else in or around” their building “every day or a few times a week.” (The difference in these two estimates of reported smoke incursion can be explained by variation in item wording.) The former value is relatively low when compared with prevalence of incursions reported in other studies of SHS exposure in multi-unit housing (ranging from 26% to 64%) [8]. Moreover, all PHA EDs in our study confirmed that enforcing smoke-free policies within housing units has been challenging because of various obstacles, such as proving that a tenant is smoking in their unit; uncertainty about whether the court system will support evictions because of tenant violations of the smoke-free policy; and the additional burden of responding to complaints about marijuana smoking, which is not covered by the policy. These concerns echo perceived barriers to implementing voluntary smoke-free building policies among multi-unit housing operators reported in past studies [8]. 

### Limitations

This study should be interpreted in light of the following limitations. First, it is possible that residents who participated in our study are different than those who chose not to participate. For example, participants may have been more favorable to smoke-free environments and policies than residents who chose not to participate, which could, in turn, affect their behavioral response to the policy. Second, most resident respondents were non-smokers, which may not be representative of the entire population; nevertheless, the reported prevalence of tobacco use in our study (16.8% at Wave 1) is similar to a recently published 15.7% smoking rate among adult NYCHA residents [16]. Third, all outcome variables were self-reported; passive air quality measurement and biomarkers of secondhand exposure were not collected. Social desirability may have influenced resident answers, particularly about following rules, and even non-smokers may not accurately report exposure to SHS [18]. Additionally, the two data collections occurred during different times of the year (July–September at wave 1 and March–June at wave 2). We do not anticipate that seasonal variations in tobacco use and weather-related impacts on one’s ability to go outside to smoke have impacted findings. March/April are significantly colder than July–September in New York state, and we would expect more reported SHS incursions in colder months due to people smoking inside [19], not fewer, as we observed in our study. Finally, as a pre-test/post-test evaluation design of an exogenous policy intervention, we are inferring the causal effect of the HUD rule on study outcomes but cannot be certain that observed changes are necessarily associated with rule implementation. By limiting our analyses to those who indicated living at their current address for at least one year, we strengthen our confidence that respondents were exposed to the change in policy status from before to after HUD rule implementation; however, similar data collected from a sample of multi-unit housing residents of properties not subject to the HUD rule would have helped strengthen our confidence in attributing changes observed to HUD rule implementation. Future studies could examine the extent to which PHA residents covered by such policies experience salutary changes in their health and well-being. Additionally, data collected at another post-implementation time point would enable evaluation of the sustainability of observed effects.

## 5. Conclusions

This study illuminated short-term positive outcomes related to SHS exposure and smoking behavior in multi-unit public housing developments subject to a comprehensive smoke-free policy. Long-term success of the rule on the health of public housing residents can be bolstered through continued education efforts for residents and staff and additional support for directors and DMs to overcome implementation challenges.

## Figures and Tables

**Table 1:** Wave 1 and Wave 2 sample characteristics.

Characteristics	Wave 1 (2017, N = 761)		Wave 2 (2019, N = 649)	
	*n*	%	*n*	%
**Age**				
18–24	8	1.1	8	1.3
25–34	41	5.4	25	4.0
35–44	77	10.2	58	9.2
45–64	252	33.5	213	33.8
65 or older	375	49.8	327	51.8
**Race**				
White	272	45.8%	232	44.9%
Black or African American	247	41.6%	205	39.7%
Asian	38	6.4%	37	7.2%
Native Hawaiian or Pacific Islander	9	1.5%	8	1.5%
American Indian or Alaska Native	12	2.0%	6	1.2%
Multi-race	16	2.7%	29	5.6%
**Ethnicity**				
Not Hispanic or Latino	388	52.6	343	54.6
Hispanic or Latino	350	47.4	285	45.4
**Sex**				
Male	210	28.2	181	28.2
Female	535	71.8	460	71.8
**Length of time in current apartment unit**				
Less than a year	30	4.0	23	3.6
1–5 years	198	26.2	133	20.7
6–10 years	141	18.7	132	20.6
More than 10 years	387	51.2	354	55.1

Note: Bolding indicates variable name.

**Table 2:** Combusted tobacco or electronic vapor product use and number of days used by residents or guests inside residential units within the past 7 days.

Variable	Wave 1 (N = 726)	Wave 2 (N = 619)
**Current Use (N (%))**		
Current tobacco use (cigarettes, cigars, pipes, or hookah)	121 (16.8%)	58 (9.5%)
Current use of electronic vapor products (e-cigarettes, e-hookah, vape pens)	23 (3.2%)	12 (2.0%)
**Number of days used (mean (SD). median, (interquartile range))**		
Days respondent smoked a tobacco product in their unit (among smokers)	4.0 (3.1), 4, (7)	2.3 (2.9), 0, (5)
Days respondent used an electronic vapor product in their unit (among vaping product users)	1.6 (2.5), 0 (2)	1.6 (2.2), 1 (2)
Days someone else smoked a tobacco product in respondent’s unit	0.4 (1.5), 0, (0)	0.2 (1.0), 0, (0)
Days someone else used an electronic vapor product in respondent’s unit	0.04 (0.5), 0, (0)	0.04 (0.5), 0, (0)

Note: Includes only respondents who reported living in their unit for at least 1 year. Bolding indicates variable name.

**Table 3:** Proportion of residents who reported smelling tobacco smoke in the past 7 days in living and common areas.

Area of Building	Wave 1 (N = 726)		Wave 2 (N = 619)	
	n	%	n	%
Any area	583	86.1	411	73.0
Shared laundry rooms	78	20.6	39	11.9
Recreation room and/or party room	83	20.9	32	9.4
Inside your apartment	215	36.6	104	19.9
Porches or patios	217	49.7	149	39.2
Lobby and/or lounge area	323	54.2	210	41.1
Indoor shared areas, like stairwells and hallways	400	59.5	273	46.8
Shared large outdoor areas, like parking lots, lawns, or playgrounds	376	65.2	226	47.5
Within 25 feet of your building	407	67.8	295	56.5

Note: Includes only respondents who report living in their unit for at least 1 year.

**Table 4:** Multivariate regression results for SHS exposure, smoke-free policy noncompliance, and presence of restrictive home smoking rules.

Explanatory Variable	Smells Smoke Anywhere in Development	Smoke Entering Unit	Respondent Smoking In-Unit (Restricted to Smokers)	Someone Else Smoking In-Unit	Restrictive Home Smoking Rules	Number of Places Smoke Smelled in Development
	N = 939	N = 1002	N = 144	N = 992	N = 1002	N = 939
	Odds Ratio					Coefficient (95% Confidence Interval)
	(95% Confidence Interval)					
**Wave (ref = Wave 1)**						
Wave 2	0.23 ** (0.13–0.44)	0.39 ** (0.23–0.67)	0.01 **(0.00–0.16)	0.45 * (0.23–0.88)	3.63 ** (2.20–6.00)	−0.70 ** (−0.93–−0.47)
**Repeat response (ref = non-repeat respondents)**						
Repeat respondents	1.51 (0.80 –2.88)	2.53 * (1.22–5.24)	9.97 (0.33–298.78)	1.17 (0.58–2.38)	1.08 (0.68–1.70)	0.18 (−0.13–0.49)
**Age (ref = 18–64)**						
Aged 65+	0.48 * (0.27–0.87)	0.22 ** (0.10–0.47)	1.56 (0.05–50.04)	0.49 (0.24–1.01)	1.60 * (1.03–2.47)	−0.59 ** (−0.88–0.30)
**Sex** (ref = female)						
Male	0.81 (0.43–1.55)	0.33 ** (0.16–0.69)	4.14 (0.19–91.73)	1.25 (0.63–2.51)	1.78 * (1.08–2.94)	−0.17 (−0.48−0.14)
**Race (ref = White)**						
Black or African American	0.80 (0.36–1.78)	1.01 (0.44–2.33)	0.21 (0.002–21.98)	0.88 (0.39–1.99)	0.80 (0.45–1.42)	0.10 (−0.30–0.50)
Asian	0.50 (0.14–1.74)	0.54 (0.14–2.15)	0.00 (0.000–570.31)	0.51 (0.10–2.60)	1.40 (0.52–3.77)	0.04 (−0.59–0.66)
Native Hawaiian, Pacific Islander, American Indian, or Alaska Native	1.62 (0.23–11.24)	8.97 * (1.66–48.63)	0.01 (0.000–12.20)	1.83 (0.35–9.52)	0.41 (0.13–1.30)	0.38 (−0.49–1.26)
Multi-race	0.65 (0.15–2.92)	1.81 (0.34–9.76)		1.95 (0.34–11.32)	0.82 (0.25–2.71)	0.43 (−0.41–1.27)
**Ethnicity (ref = not Hispanic/Latino)**						
Hispanic or Latino	1.03 (0.47–2.24)	0.69 (0.30–1.63)	0.90 (0.005–168.36)	0.29 ** (0.12–0.70)	0.93 (0.53–1.65)	0.28 (−0.11−0.67)
**Smoking status ref = non-current smoker**)						
Current smoker	1.12 (0.48–2.65)	0.32 * (0.13–0.80)		11.01 ** (3.98–30.45)	0.08 ** (0.03–0.18)	-0.41* (-0.79–−0.04)
**Constant**	37.92 ** (9.35–153.73)	17.58 ** (5.05–61.17)	251.72 (0.98–64,545.99)	0.04 ** (0.009–0.16)	2.00 * (1.03–3.91)	3.37 ** (2.90–3.83)

Note: Includes only respondents who report living in their unit for at least 1 year. All models accounted for within-unit correlation and controlled for PHA-level fixed effects (not shown in regression table). All models were conducted using logistic regression, except for the model of the number of places respondents smelled smoke within their building, which was conducted as a linear regression. For logistic regression models, we present odds ratios rather than coefficients. Due to cell size limitations, the model of respondents smoking in units did not include an indicator for model specification: outcome = β0 + β1 (Wave 2) + β2 (repeat respondent) + β3 (age 65+) + β4 (male) + β5 (current smoker) + β6-9 (PHA indicators) ** *p* < 0.01, * *p* < 0.05. Bolding indicates variable name.

## Data Availability

The data underlying this article are not publicly available due to commitments made to the participating public housing authorities. However, the data with PHA identifiers removed can be made available upon request from the authors.
